# Opinion: Auxiliary Role of Medical Imaging Technology in Clinical Surgery

**DOI:** 10.3389/fsurg.2022.913427

**Published:** 2022-05-02

**Authors:** Zhang Ziyi, Li Shuang, Zhao Shihao, Zuo Zhikai, Liu Yunrun

**Affiliations:** Hong Kong Baptist University, Kowloon, Hong Kong, SAR China

**Keywords:** medical imaging, surgery, clinical, image guided surgery (IGS), tecnology

## Introduction

Medical imaging, as a biological imaging discipline, is very effective in the current clinical surgery. This digital technology using imaging can provide doctors with accurate imaging data to help them judge patients, diseases in the entire surgery process. In recent years, through the continuous research and practice of researchers, the role and importance of medical imaging technology in clinical surgery has been improved year by year. At present, the application of digital imaging means in clinical medicine has produced many kinds of derivative technologies, such as CT, MRI, color ultrasound, DSA and other new equipment or technological innovations in the 21st century as shown in Table 1, which have been widely used in standardized medical institutions all over the world, and have achieved satisfactory results in the diagnosis of clinical diseases, making it an indispensable means in clinical medical diagnosis and treatment. The application of medical imaging technology in clinic can not only make doctors have more efficient and accurate diagnosis in clinic, but also provide more reliable diagnosis and treatment schemes for patients and improve the success rate of final surgery.

**Table 1:** Application of medical imaging in surgery.

Before surgery		In surgery	
Technology	Function	Technology	Function
Computed radiography (CR)	It can be used for general X-ray examination in radiology department, and it can make plain X-ray examination digital.	Needle guide and Image guided surgery	1. Collect medical image data of patients in real time. 2. Quickly process and analyze data. 3. Three-dimensional visualization technology can reconstruct the lesion in real time and display the structural features around the surgical field. 4. AR real-time display of surgical path. 5. Show the possible tissue structures on the surgical path. 6. Showing important tissue structures that should be avoided, such as blood vessels, nerves and bones, can show the scope of the lesion that needs treatment. 7. Show the range of patients needing treatment. 8. The position and posture of the surgical instruments can be accurately calculated and displayed in real time. 9. Display the spatial relationship between surgical instruments and tumors, and indicate and adjust the direction of surgical instruments.
Computed tomography (CT)	It can visually display the size and adjacent relationship of the lesion on the picture, and accurately judge the volume, length, distance and angle, etc.		
Digital subtraction angiography (DSA)	Applied to whole-body cardiovascular imaging.		
Magnetic resonance imaging (MRI)	It can directly make cross-sectional, sagittal, coronal and various oblique plane tomography images.		
Ultrasound-Guided (USG)	It can be used for imaging diagnosis of thoracic cavity, heart, abdominal parenchymal organs, pelvic organs, fetus, blood vessels of limbs, small organs and superficial tumors.		

Nowadays, doctors can keep a real-time understanding of the patient's affected areas at all times during the operation. And medical imaging equipment can perform modeling, feed back real-time images, AR analysis of various patient parts and other functions to assist doctors in the operation. This can not only reduce the surgical accidents caused by doctors’ inexperience in traditional surgery, but also help doctors to find the difficult-to-find pathological parts that they didn’t notice before or during the operation in time, and help doctors to better avoid nerve tissue and other non-diseased tissues through real-time medical imaging equipment during the operation, thus greatly reducing the failure rate of the operation (1). The development of medical imaging in clinical surgery also greatly reduces the burden, pressure and duration of surgery borne by doctors and patients during surgery. With the development of digitalization, networking and intelligence of modern medical imaging technology, medical imaging technology is constantly practiced and run-in on the basis of complex medical information visualization. Through computerization and virtual reality, surgery is becoming a new compound technology field to make up for the shortcomings of traditional surgery.

## Application of Medical Imaging in Surgery

### Preparatory Work Before and After Surgery

The application of modern medical imaging can provide doctors and patients with a large number of imaging materials before and after surgery, which can help doctors to fully grasp the patients’ condition. For example, the computerized X-ray photography used in preoperative and post-perative examination is one of the main auxiliary imaging methods for clinical diagnosis before and after surgery. In clinical use, according to the difference of the density and thickness of different patients’ bodies, the degree of X-ray absorption is different, which makes the lesions in patients’ bodies form different black and white images on X-ray films. However, with the appearance of CR and DR, the radiation dose is greatly reduced, which can better protect patients, reduce the adverse effects caused by radiation, and make the image clearer, helping doctors to confirm the location of the lesion faster. In before and after of the surgery, the clinical application of multi-slice spiral CT shows that it plays an important role in the differentiation of malignant tumors, and it can help doctors to accurately collect the volume, size, length and other data of the lesion site, which is convenient for later surgery and rehabilitation examination. In preoperative tumor diagnosis and postoperative examination, not only CT can help doctors to make a correct judgment on the patient's condition, but digital subtraction angiography(DSA) can also accurately diagnose the lesion. Similarly, in clinic, DSA is applied to the vascular imaging of various systems of the whole body, such as bronchial angiography, renal artery DSA, hepatic artery DSA, splenic artery DSA, lower limb DSA, etc., which is helpful to the imaging diagnosis of arteriosclerosis, venous thrombosis, varicose veins and other diseases. However, for complex areas in patients’ bodies, such as heart and pelvic cavity, USG can efficiently perform imaging examination on chest cavity, heart, abdominal parenchymal organs, pelvic organs, fetus, blood vessels of limbs, small organs and superficial tumors. In particular, USG has real-time three-dimensional imaging technology, which can be used in prenatal diagnosis of pregnant women and before operation of patients with heart diseases (2). In clinic, it can accurately judge congenital malformations such as limb ischemia, cleft lip and spina bifida, as well as heart valvular diseases, intracardiac tumors and thrombi of patients with heart diseases. Through medical imaging researchers’ research on X-ray-based imaging medicine in quantum physics, the development of magnetic resonance imaging(MRI) provides a safer and more reliable guarantee for patients’ preoperative examination and regular check-up after operation without radiation, non-invasion, intubation and contrast agent injection. Moreover, doctors can collect MRI images of patients’ diseased parts by using MR urography, MR pancreaticobiliary duct imaging, MR spinal cord imaging, MR inner ear water imaging, MR fallopian tube imaging and other technologies, and perform three-dimensional imaging on the cross section of patients’ bodies in all directions, so as to better locate the diseased parts. According to the development of these pre-operative and postoperative imaging technologies, technology is constantly developing and innovating, such as pre-operation and postoperative medical image simulation, medical image surgery success rate measurement and so on. Patients and doctors can get more efficient and low-pressure digital information before and after the operation, which is more conducive to promoting the modernization of medical level and maximizing the interests of patients (3).

### Role in Surgery

The development of modern digital medical treatment provides accurate image data for the preparation of surgery, but in most cases, doctors often need to travel back and forth between departments and image processing stations, which leads to the inability to grasp the diseased parts of patients in real time in emergency situations. Therefore, in the continuous experiments of researchers and a few clinical practices at present, medical imaging technology can analyze the patient's lesion location and other data in the body for the surgeon through real-time data transmission or accurate positioning of images during the operation. For example, holographic augmented reality navigation, which is still in the experimental and clinical pilot stage at present, realizes holographic augmented reality navigation in liver puncture surgery by means of human-computer interface design and equipment, and doctors can analyze the location of liver lesions of patients by wearing equipment (4). This medical imaging technology can greatly help doctors to observe most parts that cannot be observed with naked eyes. Different from other rigid body operations such as orthopedics, flexible body operations such as liver require more data, resulting in lower fault tolerance rate and higher risk. Thus, in this kind of surgery, the average accuracy of liver phantom puncture guided by augmented reality is 3.23 mm, which greatly improves the surgical accuracy and the success rate of patients. In some cases, with the development of holographic technology, the compatibility of informatization can be seen (5). The multi-level interactive visualization system of medical images for surgical navigation can not only rely on large medical equipment, but also be compatible with ios and other platforms. In the process of surgery, doctors can use AR equipment carried by the cutting edge of surgical instruments to measure the distance of tumor and other pathological tissues, monitor and calculate the deviation value of surgical path in real time, and calculate the minimum dangerous distance from surrounding tissues and give real-time warning. Not only the data transmitted by the tip of the instrument, but also the coordinates and markers acquired by the tip in the AR equipment worn by doctors. And the visualization module of surgical navigation, doctors can clearly understand the actual scene in the patient's body, and can observe the layered display effect brought by holographic images on the medical display (6). AR will demarcate and display different areas in the patient's body, and render and calibrate the designated special areas through the doctor's preset interest areas, and dynamically track them in real time. And the device can be used to review the patient's original tumor part to understand the recovery after the surgery. However, at present, according to the actual use effect brought by the optimization of visualization equipment and programs, a small amount of auxiliary information may be missing in the operation process, such as the wearing problem of AR glasses or the rendering speed, so many researches and designs are still in the experimental stage (7). It is believed that in the future, the intraoperative medical imaging technology can combine the AR-assisted surgical navigation module with the three-dimensional model constructed by CT, MRI and other medical data to better obtain the pose information. And three-dimensional registration is carried out, so that doctors can see the superposition effect of the virtual model and the surgical path with real patients in real time during the operation, and help them to make more intuitive judgments and perform the operation accurately (8).

## Discussion

With the help of modern medical imaging, doctors can get a lot of data about patients’ condition before and after the surgery through CT, MRI, DSA, USG and other imaging technologies. The data accuracy rate of medical imaging is increasing. According to the research and development of medical imaging technology, such as AR equipment and IGS medical instruments, intelligent imaging surgery auxiliary equipment can not only enable doctors to complete surgery and obtain auxiliary information more efficiently and quickly, but also promote the standardized development of clinical surgery today, greatly avoid surgical accidents caused by doctors’ lack of experience or information, and can help doctors better understand the recovery status of patients after surgery and provide information support for patients’ recovery after surgery. Now, the preoperative and postoperative medical imaging diagnostic equipment has been gradually improved and a complete diagnosis and treatment mechanism has been established, while the intraoperative imaging equipment is still in the development and experimental stage. However, the development prospect of medical imaging is full of opportunities (9). Advanced medical imaging diagnosis and treatment equipment is expected to solve the technical difference and the difference of operation success rate between large hospitals and small health institutions in the future, and it will also practice higher research value and more direct diagnosis and treatment effect for patients in medical imaging surgery research. In the future, medical imaging technology will continuously integrate the data cores of various disciplines, and it is also expected to rapidly move from laboratory to clinic, contributing to the development of modern medicine.
